# Anti-proliferative and pro-apoptotic effects of GHRH antagonists in prostate cancer

**DOI:** 10.18632/oncotarget.10710

**Published:** 2016-07-19

**Authors:** Laura Muñoz-Moreno, Maria Isabel Arenas, María J. Carmena, Andrew V. Schally, Manuel Sánchez-Chapado, Juan C. Prieto, Ana M. Bajo

**Affiliations:** ^1^ Department of Systems Biology, Unit of Biochemistry and Molecular Biology, University of Alcalá, Alcalá de Henares, Spain; ^2^ Department of Biomedicine and Biotechnology, Unit of Cell Biology, University of Alcalá, Alcalá de Henares, Spain; ^3^ Veterans Administration Medical Center and South Florida Veterans Affairs Foundation for Research and Education, Miami, FL, USA; ^4^ Departments of Pathology and Medicine, Division of Oncology and Hematology, University of Miami Miller School of Medicine, Miami, FL, USA; ^5^ Department of Surgery and Medical and Social Sciences, University of Alcalá, Alcalá de Henares, Spain; ^6^ Department of Urology, Príncipe de Asturias Hospital, Alcalá de Henares, Spain

**Keywords:** GHRH, GHRH antagonists, cell proliferation, apoptosis, prostate cancer therapy

## Abstract

Growth hormone-releasing hormone (GHRH) and its receptors have been implicated in the progression of various tumors. *In vitro* and *in vivo* studies have demonstrated that GHRH antagonists inhibit the growth of several cancers. GHRH antagonists, JMR-132 and JV-1-38 inhibit the growth of androgen-independent prostate tumors. Here we investigated the involvement of GHRH antagonists in proliferative and apoptotic processes. We used non-tumoral RWPE-1 and tumoral LNCaP and PC3 human prostatic epithelial cells, as well as an experimental model of human tumor PC3 cells. We evaluated the effects of JMR-132 and JV-1-38 antagonists on cell viability and proliferation in the three cell lines by means of MTT and BrdU assays, respectively, as well as on cell cycle and apoptotic process in PC3 cells. The expression levels of PCNA, p53, p21, CD44, Cyclin D1, c-myc, Bax and Bcl2 were determined in both *in vivo* and *in vitro* models by means of Western-blot and RT-PCR. GHRH antagonists suppressed cell proliferation and decreased the levels of the proliferation marker, PCNA, in the three cell lines and in PC3 tumor. GHRH antagonists led to an increase of cells in S-phase and a decrease in G1 and G2/M phases, and induced S-phase arrest and increase of apoptotic cells. The effects of GHRH-antagonists on cell cycle could be due to the changes observed in the expression of p21, p53, Bax, Bcl2, CD44, Cyclin D1, c-myc and caspase 3. Present results confirm and extend the role of GHRH antagonists as anti-proliferative and pro-apoptotic molecules in prostate cancer.

## INTRODUCTION

Prostate cancer is the second most common malignancy worldwide, and one of the most common cancer-specific cause of mortality in men [1]. Currently, treatment modalities for this disease in a localized stage or when it is still castration-sensitive yield good results in most patients. However, such treatments are only palliative in the advanced stage [2]. Therefore, new therapeutic targets should be found in order to get effective treatments for more aggressive stages in prostate cancer.

Growth hormone releasing-hormone (GHRH) is a hypothalamic neuropeptide that regulates the secretion of growth hormone (GH) from the anterior pituitary after binding to its receptor (GHRH-R) [3], which belongs to the family of G protein-coupled receptors (GPCRs) [4]. Besides the pituitary type of GHRH receptors (pGHRH-R), four splice variants have been described [5, 6], of which SV1 is the main isoform. GH stimulates the production of insulin-like growth factor I (IGF-1) from the liver and other tissues. IGF-1 plays a crucial role on the progression of various tumors [4]. The pGHRH-R and SV1 mediate the stimulatory effects of GHRH and the inhibitory effects of GHRH antagonists on tumor tissues [5, 7].

The group of one of us (AVS) has synthesized numerous GHRH antagonists with potential therapeutic usefulness in the management of various tumors. Various *in vitro* and *in vivo* studies demonstrated that several GHRH antagonists suppress the growth and enhance apoptotic processes in prostate cancer and other experimental cancers [8–11]. In previous studies, we reported that the GHRH antagonists, JMR-132 and JV-1-38, significantly reduce tumor proliferation in mice xenografted with PC3 prostate cancer cells [12]. In addition, we have described that a peptide structurally related to GHRH, vasoactive intestinal peptide (VIP), increases the proliferation and regulates the expression of specific markers in prostate cells [13].

The control of cell proliferation is essential to maintain tissue homeostasis. When such control fails, uncontrolled proliferation of cells may contribute to initiation of the carcinogenic process. Balance between cell proliferation and death is crucial in controlling tumor progression [14]. In this regard, cell cycle and apoptosis are responsible for regulating cell number and removing damaged cells. Numerous molecules interact with the different proteins involved in cell cycle modulation including the proliferating cell nuclear antigen (PCNA), which acts as a processing factor for DNA polymerase during DNA replication [15, 16]. On the other hand, p21 protein, a cyclin-dependent kinase (CDK) inhibitor, is capable of binding to both cyclin-CDK and PCNA. Through its binding to PCNA, p21 inhibits replication by blocking the ability of PCNA to stimulate DNA polymerases [17], and leads to cell growth arrest in the mitotic cycle [18]. The antiproliferative actions of p21 may occur by a p53-dependent mechanism [19]. In addition, p53 induces apoptosis through the regulation of apoptotic genes. In this context, p53 activates and represses the transcription of Bax (pro-apoptotic) and Bcl2 (anti–apoptotic), respectively, leading to activation of the programmed cell death process [20].

The aim of this study was to determine the effects of GHRH antagonists, JMR-132 and JV-1-38, on different processes such as proliferation, apoptosis and cell cycle involved in the progression of prostate cancer in an experimental model of androgen-independent cell PC3 tumors and prostate tumor cell lines.

## RESULTS

### Effect of GHRH and its antagonists on cell viability and cell proliferation in RWPE-1, LNCaP and PC3 cells

**Figure 1:**
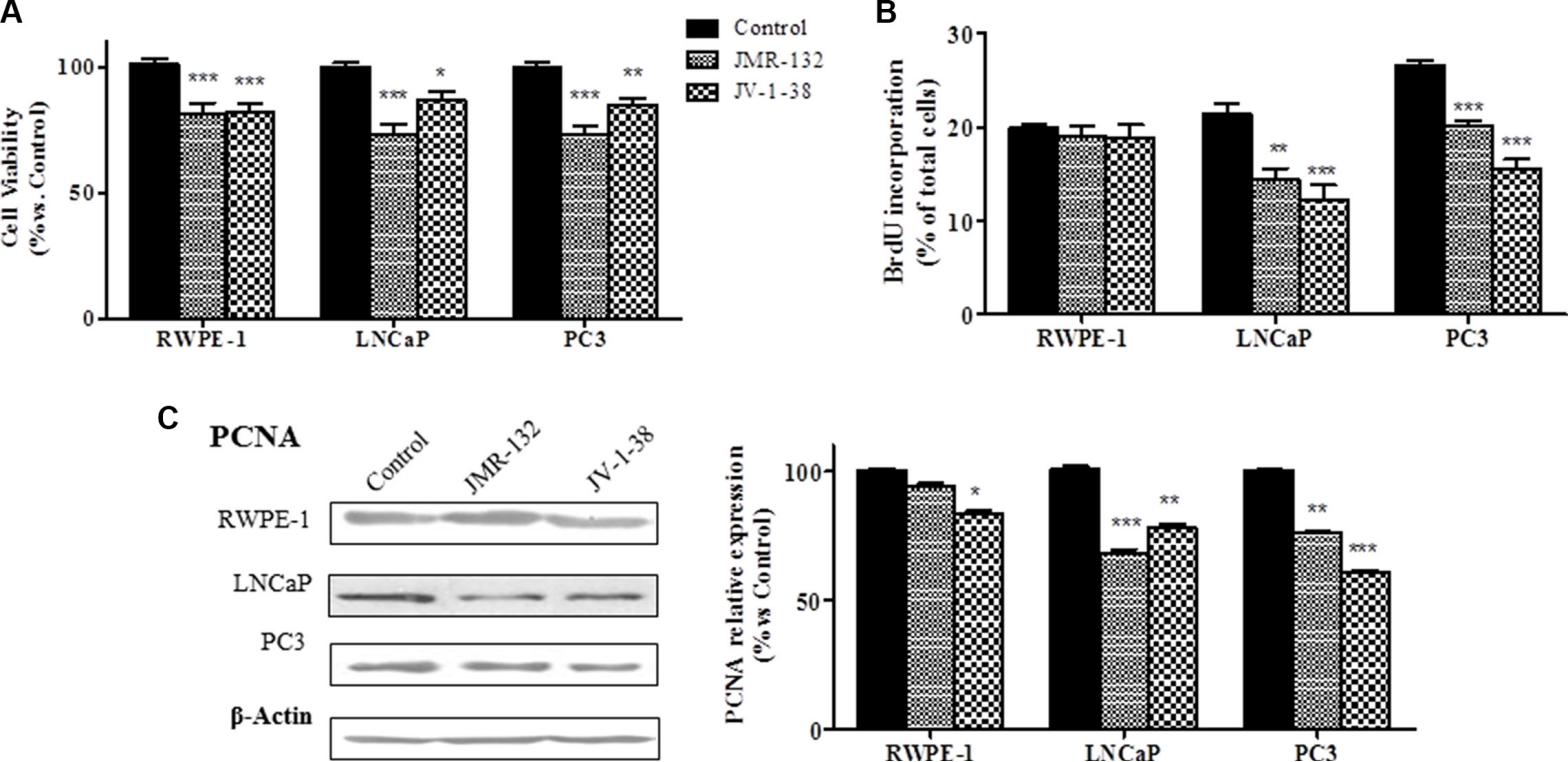
Effect of GHRH antagonists, JMR-132 and JV-1-38, (0.1 μM) on cell viability (A), cell proliferation (B) and expression of PCNA in RWPE-1, LNCaP and PC3 cells The outcome was evaluated by means of MTT (A), BrdU incorporation (B) assays and Western blot assays (**C**). The results are expressed as percentage of control value. Data are mean ± SEM of ten independent experiments; *p < 0.05; **p < 0.01; ***p < 0.001.

The effect of GHRH antagonists on cell viability of RWPE-1, LNCaP and PC3 cells was assessed by MTT assays (Figure 1A). Treatment with 0.1 μM GHRH antagonists significantly decreased the viability in all cell types (by 20–28% vs control). In order to compare the effect of GHRH and its antagonists on cell proliferation, BrdU incorporation assays were performed in the three cell lines (Figure 1B). GHRH antagonists showed no effect in RWPE-1 cells. However, in LNCaP and PC3 cells, JMR-132 and JV-1-38 provoked a significantly reduction of proliferation (by 25–47% vs control), with a greater effect in PC3 cells.

Changes in cell proliferation induced by GHRH antagonists may be due to variations on the expression of molecules such as PCNA. We studied whether GHRH antagonists modify the expression of PCNA at 8 h after treatment (Figure 1C). In RWPE-1 cells, JV-1-38 only significantly reduced the expression of PCNA, but the treatment with JMR-132 did not produce changes. However, in LNCaP and PC3 cells both GHRH antagonists decreased the expression levels of PCNA (by 25–40% vs control).

### Effect of GHRH antagonists on cell cycle and apoptosis in PC3 cells

**Figure 2:**
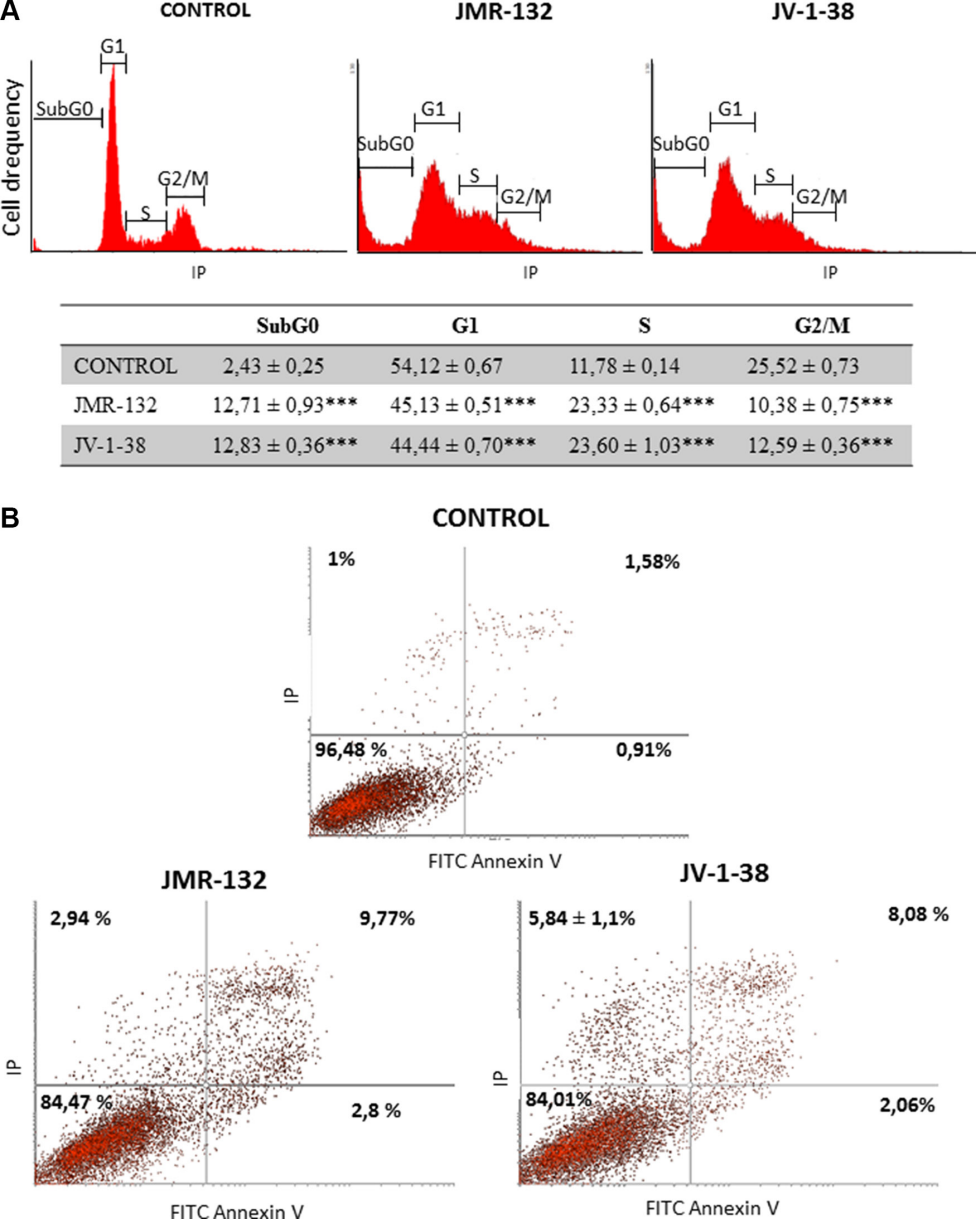
Analysis of cell cycle (A) and apoptosis (B) in PC3 cells after treatment with GHRH antagonists JMR-132 or JV-1-38 Cells were treated for 8 h (A) and 24 h (B) with 0.1 μM GHRH antagonists. The results are shown as percentage of cells in each phase of the cycle (A) and, early (Lower right) and late (Upper Right) apoptotic cells (B), as compared to untreated control cells. A representative experiment is shown. Data in the table are the means ± SEM of four independent experiments; ***p < 0.001 vs. control.

GHRH antagonists showed the greatest effects on both viability and proliferation in PC3 cells, which represent a highly aggressive stage in prostate carcinomas. In order to investigate whether such an effect is due to changes on cell cycle, we analyzed the DNA content in PC3 cells. It reveals the distribution of cells in three major phases of the cycle and makes it possible to detect apoptotic cells with fractional DNA content. Thus, SubG_0_ phase determines whether the DNA has been damaged and the genetic material is hypodiploid (< 2n). Cell cycle results are shown in Figure 2A. After treatment with GHRH antagonists, there were a large number of cells in SubG_0_ phase revealing an increase in apoptotic cells. Moreover, GHRH antagonists caused a notable increase of cells in S-phase and a corresponding decrease of those in the G1 and G2/M phases.

Moreover, we investigated whether the GHRH antagonists provoke changes in apoptotic process by staining of cells with annexin V and PI. Figure 2B shows the changes in percentage of cells after 24 h with GHRH-antagonists treatment compared to the control group. JMR-132 and JV-1-38 significantly increased the percentage of early (lower right) and late (upper right) apoptotic cells, by 1–1.9% and 6.5–8.2%, respectively.

### Effect of GHRH antagonists on p53, p21, CD44, c-myc, cyclin D1 and Bax/Bcl2 expression in PC3 cells

**Figure 3:**
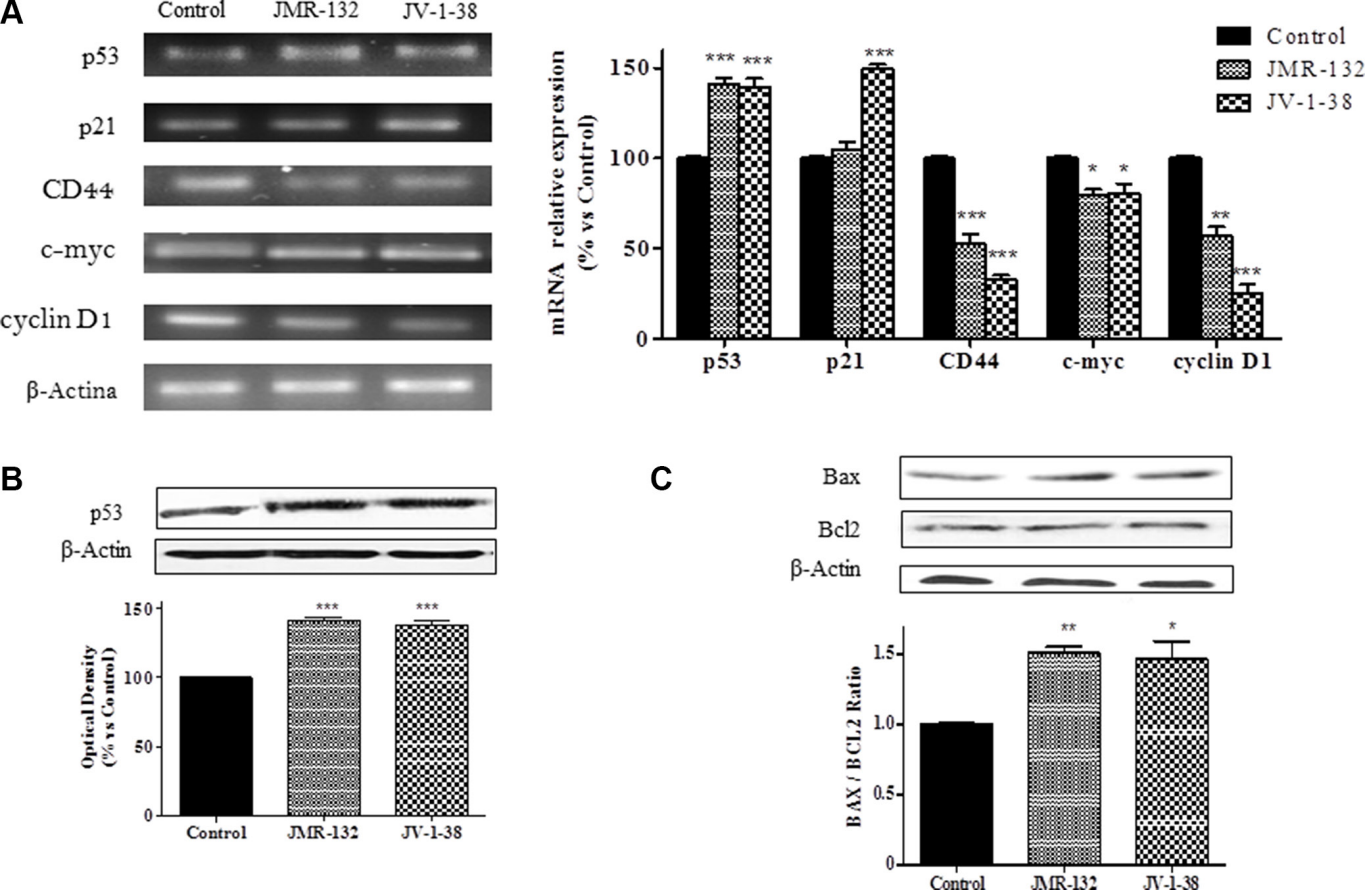
Analysis of p53, p21, CD44, c-myc and cyclin D1 mRNA levels (A) and p53 (B), Bax and Bcl-2 (C) protein levels after treatment with 0.1 μM of GHRH antagonists at 30 min (A), 60 min (B), and 2 h (C) respectively, in PC3 cells The study was performed by RT-PCR and Western-blot assays. The results are expressed as percentage of control value. Data are mean ± SEM of four independent experiments; *p < 0.05; **p < 0.01; ***p < 0.001.

Changes in cell cycle, apoptosis and proliferation induced by GHRH antagonists may be due to variations on the expression of molecules involved in this process. In this context, we analyzed the levels of p53, p21, CD44, c-myc and cyclin D1 in PC3 cells. Treatment with GHRH antagonists increased p53 mRNA levels up to 40% after 30 min (Figure 3A). These results agreed with a significant increase (by 40%) of p53 protein expression at 60 min after treatment with GHRH antagonists (Figure 3B). Furthermore, JV-1-38 provoked a significant increase of p21 mRNA levels (by 50%) after 2 h of treatment (Figure 3A). On the other hand, GHRH antagonists decreased CD44, c-myc and cyclin D1 mRNA levels after 2 h of treatment (Figure 3A).

In order to determine whether the apoptosis observed after treatment with GHRH antagonists in PC3 cells was related to variations on the levels of molecules involved in apoptosis, Bax/Bcl2 ratios were studied. After 2 h of treatment, GHRH antagonists provoked a decrease on the expression of Bcl2 and an increase on that of Bax, leading to an increase in cell apoptosis (Figure 3C).

### Effect of GHRH antagonists, JMR-132 and JV-1-38 on tumor weights from xenografted PC3 prostate cancers

Both GHRH antagonists reduced the weight of PC3 tumors in nude mice. After 41 days of treatment, JV-1-38 administered at 20 μg·day^−1^ per animal significantly (*P* < 0.05) reduced the mean tumor weight to 270 ± 102 mg compared with that in the control group (756 ± 101 mg), corresponding to a decrease of about 64% (Table 1). A greater reduction in tumor weight (69%, *P* < 0.05) was observed in mice treated with JMR-132 at 10 μg·day^−1^ per animal.

**Table 1:** Effect of treatment with GHRH antagonists JMR-132 (10 μg/day) and JV-1-38 (20 μg/day) on tumor weight and on tumor burden in PC3 cells xenografted into nude mice

Treatment groups and the number of evaluable animals	Tumor weight, mg (% inhibition)	Tumor burden, μg/g body weight (% inhibition)
Control (*n* = 10)	756 ± 101	24.59 ± 3.26
JMR-132 (*n* = 6)	235 ± 140 (68)*	10.15 ± 4.08 (58)*
JV-1-38 (*n* = 6)	270 ± 102 (64)*	8.40 ± 3.08 (66)*

Values are mean ± SE.

* *P* < 0.05 vs. Control.

**Figure 4:**
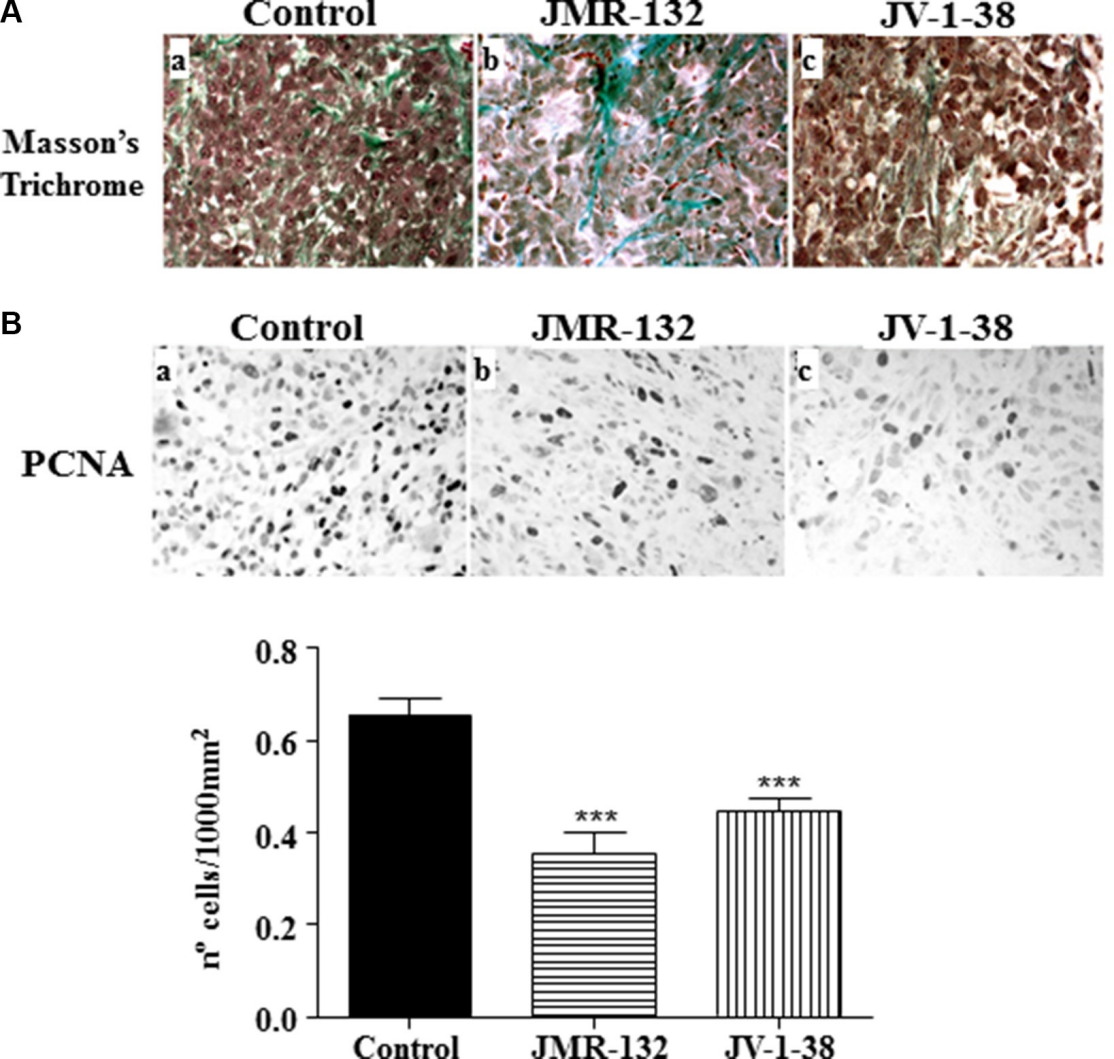
Effect of the GHRH antagonists JMR-132 (10 μg/day) and JV-1-38 (20 μg/day) on PC3 tumor proliferation (**A**) Histological sections from tumors stained with Masson's Trichrome. Control tumors presented scarce connective tissue between tumor cells (**a**); however, tumors from xenografts (**b**, **c**) presented more ground substance and areas of collagen fibers bundles. Original magnification X300. (**B**) Immunohistochemistry using a specific antibody against PCNA was performed. The highest number of proliferating cells was observed in control samples (black nuclei). Original magnification X300. The evaluation of the number of PCNA-immunoreactive nuclei is shown on graphical representation. Data in each bar are the means ± SEM. ***p < 0.001 vs. control.

The histological study of the mouse tumors showed that they were surrounded by a capsule of dense connective tissue. These masses were formed by different cell types such as large tumor cells with polygonal outline intermingled with smaller cells. In some areas, abundant lymphocyte infiltration was seen. The ground substance was scarce and some collagen fibers bundles could be observed (Figure 4Aa). The same pattern was found in both groups of xenografts from mice treated with JV-1-38 or JMR-132 except that the ground substance was more abundant than in controls (Figure 4Ab–4Ac). It would indicate the presence of a few tumor cells and a loss of adhesion between them.

### Effect of GHRH antagonists, JMR-132 and JV-1-38, on growth of xenografts of PC3 human prostate cancer cells

We analyzed the expression of cell proliferation markers such as PCNA by immunohistochemistry (Figure 4B). Control samples showed an increased number of PCNA-immunoreactive nuclei compared with xenografts from antagonist-treated mice. The median value of proliferation number for each group was 0.64 ± 0.03 for control, 0.40 ± 0.05 for JMR-132 and 0.49 ± 0.03 for JV-1-38. Overall, there were statistical differences between the control group and JMR-132 and JV-1-38 (*P* < 0.001).

### Effects of GHRH antagonists on the expression of molecules implicated in tumor proliferation and apoptotic process in PC3 tumors

**Figure 5:**
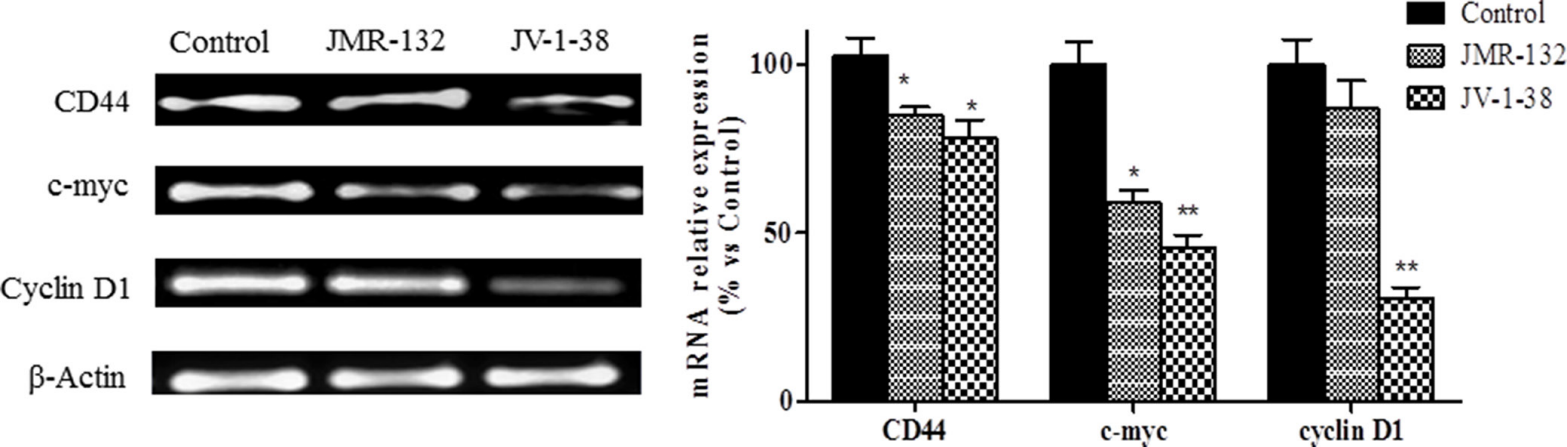
Effect of GHRH antagonists JMR-132 (10 μg/day) and JV-1-38 (20 μg/day) on the expression of different molecules involved in proliferation in PC3 tumors CD44, c-myc and Cyclin D1 mRNA levels were evaluated by RT-PCR. Expression amounts were normalized with those for β-actin. Data in each bar are the means ± SEM. *p < 0.05; **p < 0.01 vs. control.

**Figure 6:**
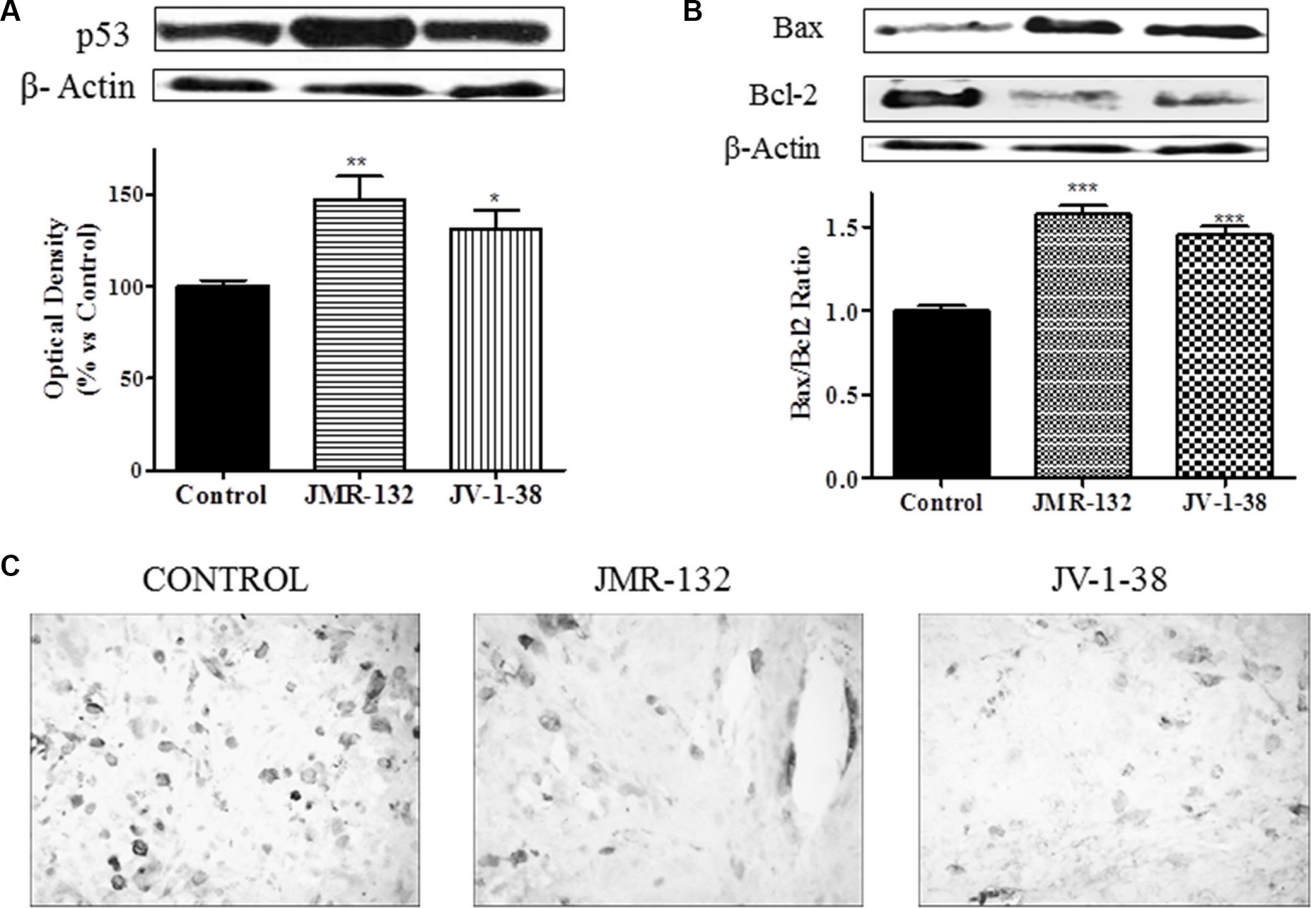
Effect of JMR-132 (10 μg/day) and JV-1-38 (20 μg/day) on the expression of different molecules involved in apoptosis and cell cycle in PC3 tumors The p53 (**A**) and Bax and Bcl-2 (**B**) protein levels were determined by Western blotting using specific antibodies. Bax/Bcl2 ratio was determined and is also shown. Immunohistochemistry using a specific antibody against Caspase 3 was performed (**C**). The highest number of apoptotic cells was observed in treatment control samples (black cells). Original magnification X300. Data in each bar are the means ± SEM. *p < 0.05; **p < 0.01; ***p < 0.001.vs. control.

We studied in PC3 xenograft material the effects of GHRH antagonists on molecules related to proliferation, cell cycle and apoptosis including CD44, c-myc, cyclin D1, p53, Bax/Bcl2 ratio and caspase 3 (Figures 5 and 6). The treatment with JV-1-38 significantly decreased (by 70%) mRNA levels for a major regulator of cell cycle, cyclin D1 (Figure 5). In addition, RT-PCR assays showed lower c-myc levels in both groups treated with antagonist, JMR-132 (by 34%) and JV-1-38 (by 51%) as compared with control group ((Figure 5). CD44 mRNA levels were significantly diminished in the groups treated with JMR-132 (by 17%) and JV-1-38 (by 24%) as compared with control group (Figure 5). Densitometry analysis of p53 revealed a significant increase on protein expression levels from tumors treated with GHRH antagonists, JMR-132 (by 47%) and JV-1-38 (by 31%) (Figure 6A).

In order to investigate the effect of GHRH antagonists on downstream targets such as the apoptotic markers Bcl-2 and Bax, Western blot assays were performed in PC3 (Figure 6B). Densitometry of the bands showed that there was a significant increase of the proapoptotic protein Bax after treatment with JMR-132 (by 18%) and JV-1-38 (by 19%) as compared with control group. However, the expression of the antiapoptotic protein Bcl-2 was significantly decreased by 25% in JMR-132 group and 17% in JV-1-38 group as compared with control group. In order to show the proapoptotic/antiapoptotic balance, the ratio Bax/Bcl2 was determined (Figure 6B). Moreover, the expression of caspase 3 was significantly reduced in JMR-132 and JV-1-38 groups (Figure 6C).

## DISCUSSION

In the current study, we investigated the effect of GHRH antagonists on proliferation and apoptosis in human prostate non-tumor and tumor cells and an experimental model of advanced prostate cancer. Other studies have shown that GHRH antagonists strongly inhibited the growth of various human experimental tumors [21]. In this regard, we evaluated the effect of JMR-132 and JV-1-38 on the viability of prostate cells by testing metabolic activity with MTT assays. The results confirmed that GHRH antagonists significantly decreased viability in the three cell lines studied. A reduction of viability has been described in breast and prostate tumor cells with the antagonist JMR-132 [22, 23] as well as in other cell types with other GHRH antagonists [10, 24–27]. Since the variations in metabolic activity provoked by GHRH antagonists may be due to mitogenic changes, we performed proliferation assays on the three cell lines. Treatment with GHRH antagonists reduced proliferation only in tumor cell lines, without changes in non-tumor cells. Therefore, these results could indicate a selective action of GHRH antagonists on prostate cancer cells. Other studies have previously described a decrease on proliferation in breast and colon cancer cells [28, 29], but the effect of GHRH antagonists was unknown in non-tumor cells. In this context, our results showed a reduction in tumor weight from mice treated with JMR-132 or JV-1-38. In a previous study, our group reported that the antagonists JMR-132 and JV-1-38 significantly reduced the tumor volume in mice xenografts with PC3 cells [12]. Taken together, these results could be attributed to a blockade of the effect of endogenous GHRH and/or to an independent mechanism of action exercised by GHRH antagonists.

The reduction of the proliferation on prostate cancer cells and tumor weight from xenografted PC3 may be reflected on the expression levels of PCNA. This fact could be due that a more active metabolism in tumor cells than that in non-tumor cells. PCNA is used as a prognostic marker of cell proliferation in cancer transformations [30]. It has been described that GHRH antagonists also reduce the expression of PCNA in benign prostatic hyperplasia, breast and cervical cancer and non-small cell lung carcinoma cells [26, 31]. GHRH antagonists caused a significant decrease in the expression levels of PCNA in prostate cancer cells and the observed tumor growth inhibition was associated with such a decrease on protein levels. Current results may provide new data about the mechanism of action of GHRH antagonists on the expression of this important regulator of cell proliferation in different experimental models.

GHRH antagonist, JMR-132 causes DNA damage in colon cancer cells, which in turn activates cell-cycle machinery arrest and cell apoptosis through the activation of p53, p21 and Bax, and the suppression of Bcl-2 [29, 32]. The tumor-suppressor protein p53 acts as a major defense against cancer and can elicit apoptotic death, cell cycle arrest, or senescence through differential activation of target genes [33]. Moreover, Bcl-2 and Bax are markers proteins of antiapoptotic and proapoptotic processes, respectively [34]. In this regard, it is recognized that disruption of apoptosis may aid tumor cells to acquire the ability to survive and initiate tumor metastasis process [35, 36]. Another regulatory protein, Cyclin D1, promotes progression of the cell cycle and its role in tumorigenesis has been demonstrated [33]. The gene c-myc and CD44, frequently altered in human cancers, emerges as oncogenic transcription factors that integrates the cell cycle with cell adhesion, and the apoptotic pathways [37–39]. The effect observed of GHRH antagonists observed on cell cycle correlates with the findings from cell proliferation assays as well as with the tumor reduction observed in advanced prostate cancer. Treatment with GHRH antagonists revealed more apoptotic cells and cell cycle arrest in S phase. Furthermore, GHRH antagonists were able to modulate molecules such as p53, p21, CD44, c-myc, cyclin D1 and Bax/Bcl2 in xenografted tumor and tumor cells triggering the starting of apoptosis and cell cycle arrest. The levels of cyclin D1, c-myc and CD44 expression decreased after treatment with both GHRH antagonists. Tumors treated with GHRH antagonist showed a decreased expression of c-myc, CD44 and Caspase 3. These results, taken together, could indicate a possible role in arresting the mitotic cell cycle and the activation of the apoptotic process.

In summary, this study shows no effect of GHRH antagonists on proliferation of non-tumor cells and provides a further elucidation of the mechanisms of action of GHRH antagonists, JMR-132 and JV-1-38, on the growth of human androgen-independent PC3 prostate cancers. The tumor-inhibitory effect of GHRH antagonists seems to involve complex mechanisms. After binding, GHRH antagonists block the stimulatory effects of autocrine/paracrine GHRH on tumors. Consequently, GHRH antagonists could be considered good candidates in the design of new therapies in advanced prostate cancer.

## MATERIALS AND METHODS

### Peptides

GHRH antagonists JV-1-38 and JMR-132 were synthesized in the laboratories of one of us (AVS). JV-1-38 and JMR-132 structures are [PhAc-Tyr^1^, D-Arg^2^, Phe(4-Cl)^6^, Har^9^, Tyr(Me)^10^, Abu^15^, Nle^27^, Har^29^] human GHRH_1-29_NH_2_ and [PhAc^0^-Tyr^1^, D-Arg^2^, Phe(4-Cl)^6^, Ala^8^, Har^9^, Tyr(Me)^10^, His^11^, Abu^15^, His^20^, Nle^27^, D-Arg^28^, Har^29^] human GHRH_1-29_NH_2_, respectively. Abu is α -aminobutyric acid, Har is homoarginine, Nle is norleucine, PhAc is phenylacetyl and Tyr(Me) is o-methyltyrosine [28, 40].

### Cell culture

Three human prostate cell lines were used. Cell lines were obtained from the American Type Culture Collection. Non-neoplastic, immortalized adult human prostatic epithelial cells, RWPE-1 (passages 3–10, ATCC CRL-11609, certified by STRS analysis), are androgen-responsive and show many characteristics of non- tumoral cells. The two human prostate cancer cell lines used exhibit different features of prostate cancer progression from early stages to androgen independence. LNCaP (passages 5–16, ATCC CRL-1740, certified by STRS analysis) is an androgen-responsive cancer cell line and PC3 (passages 5–16, ATCC CRL-1435, certified by STRS analysis) is an androgen-unresponsive cell line that may be analogous to recurrent prostate cancers that have achieved androgen independence. RWPE-1 cells were maintained in complete keratinocyte serum-free medium containing 50 μg/ml bovine pituitary extract and 5 ng/ml human epidermal growth factor (EGF). LNCaP and PC3 cells were grown and maintained in RPMI-1640 medium supplemented with 10% fetal bovine serum (FBS). All culture media contained 1% penicillin/streptomycin/amphotericin B (Life Technologies, Carlsbad, CA, USA). Culture was carried out in a humidified 5% CO_2_ environment at 37°C. After the cells reached 70–80% confluence, they were washed with PBS, detached with 0.25% trypsin/0.2% EDTA, and seeded in 24-well plates at 30,000–40,000 cells/cm^2^. The culture medium was changed every 3 days.

### Animals, xenografts and processing of tumors

Athymic male nude mice (nu/nu) 5–6 weeks old were obtained from Harlan (Oxon, UK) and maintained in microisolator units on a standard sterilizable diet. Mice were housed under humidity- and temperature-controlled conditions, and the light/dark cycle was set at 12 h intervals. Experimental procedures were carried out according to Spanish Law 32/2007, Spanish Royal Decree 1201/2005, European Directive 609/86/CEE and European Convention of Council of Europe ETS 123. Furthermore, the animals were studied under protocols approved by the Institutional Animal Care and Use Committee of the University of Alcalá. The androgen-unresponsive cell line PC3 was obtained from the American Type Culture Collection (Manassas, VA, USA) and may be related to recurrent prostate cancers that have achieved androgen independence. For preparation of xenografts, PC3 cells were mixed with Matrigel (Becton Dickinson, Madrid, Spain) synthetic basement membrane (1:1, v/v) and then injected subcutaneously into the right flank of nude mice (5 × 10^6^ cells/mouse). The experiment was started when the tumors had grown to about 75 mm^3^. Animal were randomly divided into three treatment groups: group 1 (ten mice), control, vehicle solution; group 2 (six mice), JMR-132 subcutaneously injected once a day at a dose of 10 μg/animal, and group 3 (six mice), JV-1-38, subcutaneously injected every day at a dose of 20 μg/animal. These doses of GHRH antagonists were chosen in order to obtain similar responses. The experiment was ended on day 41. After mice were anaesthetized with halothane, tumors were dissected, cleaned, and weighed. Tumor specimens were divided into three approximately equal portions: one portion was processed for immunohistochemistry (fixed in 10% formalin and paraffin embedded), and the other two portions were frozen in liquid nitrogen and maintained at −80°C for further experiments.

### Cell viability studies

Cells were grown to 70–80% confluence, harvested with trypsin/EDTA solution and seeded at low concentration (50,000 cells per well) in 24-well plates for 24 h. The culture medium was then removed and replaced with RPMI-1640 medium containing 1% antibiotic/antimycotic (penicillin/streptomycin/amphotericin B) and 0% FBS for 24 h. Cells were treated for 24 h with 0.1 μM GHRH antagonists. Cell viability was determined by tetrazolium assay, which measures the reduction of substrate MTT [3-(4,5-dimethylthiazol-2-yl)2,5-diphenyltetrazolium bromide] to a dark blue formazan product by mitochondrial dehydrogenases in living cells. MTT (5 mg/ml) (Sigma) was added to each well and the mixture incubated for 3 h at 37°C in darkness. The medium was replaced and the dark blue formazan precipitate was dissolved with 0.2 N HCl in isopropanol. Absorbance was read at 570 nm in a plate reader (ELX 800, Bio-Tek Instruments, Winooski, VT). Results were expressed as the relative percentage of absorbance compared with control cells.

### Cell proliferation assays

PC-3 (2 × 10^5^) cells were grown in 6-well plates. After 24 h, the culture medium was removed and replaced with RPMI-1640 medium containing 0% FBS and 1% antibiotic/antimycotic (penicillin/streptomycin/amphotericin B) for 16 h. Then, cells were subjected for 24 h to different treatments. In the last 30 min of incubation, cells were pulsed with 10 μM bromodeoxyuridine (BrdU). Thereafter, cells were washed with PBS, fixed with ice-cold absolute ethanol, and stored at −20°C for 30 min. Fixative was removed by centrifugation and the cell pellets were washed with PBS. DNA was partially denatured by incubation with 1 M HCl for 30 min at room temperature and then the cells were washed three times with PBS containing 0.05% Tween-20 (pH 7.4) and 0.1% BSA. Cells were incubated with 20 μl of anti-BrdU monoclonal antibody with FITC (BD Bioscience) in the dark for another 30 min period. In preparation for flow cytometry analysis, the cells were stained with propidium iodide (PI) staining solution: 50 μg/ml PI, 10 μg/ml RNase, PBS. The number of BrdU-positive cells was counted with the use of the Cyflogic program (Version 1.2.1.).

### Cell cycle assays

PC-3 (2 × 10^5^) cells were grown in 6-well plates. After 24 h, the culture medium was removed and replaced with RPMI-1640 medium containing 0% FBS and 1% antibiotic/antimycotic (penicillin/streptomycin/amphotericin B) for 16 h. After that, cells were subjected to the various treatments for 6 h. Then the cells were washed with PBS and detached with 0.25% trypsin/0.2% EDTA. The cells were centrifuged at 500 × g for 5 min at 4°C and the pellets were mixed with ice-cold 70% ethanol and then kept at −20°C for 30 min. After removing the ethanol by centrifugation, the pellets were washed with PBS and centrifuged again. The supernatants were discarded and the pellets suspended in PBS, 0.2 mg/ml RNase A and 20 μg/ml PI before flow cytometry analysis with a FACSCalibur cytometer (Becton Dickinson, San Agustin de Guadalix, Spain). Results obtained were analyzed with the Cyflogic v 1.2.1 program.

### Assessment of apoptosis by annexin V labeling

Apoptotic cell death was measured using a FITC-conjugated annexin V/PI assay kit (Beckton Dickinson) by means of flow cytometry. Briefly, 1 × 10^5^ cells were washed with ice-cold PBS, resuspended in 100 μl binding buffer, and stained with 5 μl of FITC-conjugated annexin V and 5 μl of PI. The cells were incubated for 15 min at room temperature in the dark, 400 μl of binding buffer was added, and the cells were analyzed with FACSCalibur cytometer. Results were analyzed with the Cyflogic v 1.2.1 program. The PC3 cells were gated separately according to their granularity and size on forward scatter (FSC) versus Side Scatter (SSC) plots. Early and late apoptosis were evaluated by plots with propidium iodide versus annexin. Cells only stained with annexin V were evaluated as being in early apoptosis; cells stained with both annexin V and propidium iodide were evaluated as being in late apoptosis or in a necrotic stage.

### Isolation of cell lysates

RWPE-1, LNCaP and PC3 cells (1.5–3 × 10^6^ cells) were washed with ice-cold PBS and then harvested, scraped into ice-cold PBS, and pelleted by centrifugation at 500 × g for 5 min at 4°C. For preparation of cell lysates, cells were kept on ice for 30 min in a solution containing 20 mM Tris–HCl (pH 7.5), 1 mM EDTA, 0.5 M NaCl, 1 mM EDTA, 2 mM PMSF, 5 μg/ml aprotinin, 5 μg/ml leupeptin, 5 μg/ml pepstatin. Thereafter, cells were pelleted by centrifugation at 4,000 × g for 5 min at 4°C.

### Western blot assays

The proteins from cell lysates (30 μg) extracts were denatured by heating. Then, they were resolved by 10% SDS-PAGE, and blotted onto a nitrocellulose membrane (BioTrace/NT) overnight in 50 mM Tris–HCl, 380 mM glycine, 0.1% SDS, and 20% methanol. Rabbit anti-PCNA (Life Technologies) (1:20,000), anti-p53 (Sigma-Aldrich) (1:2,000), anti-Bax or anti-Bcl-2 (Santa Cruz Technologies) (1:1,000) antibodies were then added and incubated for 1 h at room temperature. After treatment for 1 h at room temperature with the corresponding secondary antiserum (1:4,000 for anti-rabbit or anti-mouse sera), the signals were detected with enhanced chemiluminescence reagent (Pierce) using β-actin antibody as loading control.

### RNA isolation and RT-PCR

PC3 cells were placed in 6-well plates (15 × 10^3^ cells) and incubated with the different treatments in serum medium for different periods of time. Total RNA was isolated with Tri-Reagent (Sigma) according to the instructions of the manufacturer. Two μg of total RNA were reverse-transcribed using 6 μg of hexamer random primer and 200 U M-MLV RT (Life Technologies) in the buffer supplied with the enzyme, supplemented with 1.6 μg/ml oligo dT, 10 nM dithiothreitol (DTT), 40 U RNasin (Promega Madison, WI, USA), and 0.5mM deoxyribonucleotides (dNTPs). Two μl of the RT reaction were used for each PCR amplification with a primer set which amplifies cDNAs for human cysteine-rich protein with p53 or β-actin. The corresponding sequences of oligonucleotide primers were: p53 (sense 5′-AGG CCT TGG AAC TCA AGG AT-3′ and antisense 5′-TGA GTC AGG CCT TCT GTC T-3′); p21/WAF1 (sense 5′-ATG AAA TTC ACC CCC TTT CC-3′ and antisense 5′-AGG TGA GGG GAC TCC AAA GT-3′); CD44: 5′-AAGGTGGAGCAAACACAACC-3′ (sense), 5′-ACTGCAATGCAAACTGCAAG-3′ (antisense); Cyclin D1: 5′-TTCGGGATGATTGGAATAGC-3′ (sense), 5′-TGTGAGCTGGTTCATTGAG-3′ (antisense); c-Myc: 5′-AGCGACTCTGAGGAGGAACA-3′ (sense), 5′-CTCTGACCT TTTGCCAGGAG-3′ (antisense); and β-actin (sense 5′-AGA AGG ATT CCT ATG TGG GCG-3′ and antisense 5′-CAT GTC GTC CCA GTT GGT GAC-3′). PCR-conditions were: denaturation at 94°C for 5 min, followed by 26–40 cycles of 95°C for 1 min, 57°C for 1 min, 72°C for 1 min, and then a final cycle of 10 min at 72°C. The signals were normalized with the β-actin gene expression level. The PCR products were separated by electrophoresis and visualized in 2% agarose gels, stained with GelRedTM nucleic acid gel stain (Biotium, Hayward, CA, USA) and visualized under UV light.

### Histological assays

Serial tissue sections, 5-μm-thick, were deparaffinised in xylene and rehydrated using graded ethanol concentrations. In order to perform Masson' Trichrome, the sections were subjected to staining with Weigert's iron hematoxylin, for 5 min, followed by washing in water for 4 min. Cuts were staining with 33% Fuchsine acid and 66% Ponceau, for 8 min, and rinsed in water with 1% acetic acid. Then, the sections were placed in a solution of Orange G for 5 min, returning to rinse in water with 1% acetic acid, and soaked in a solution of green light (0.2% green light and 0.2% acetic acid), again washed after 3 min. Finally, become dehydrated and mounted in Entellan (Merck, Darmstadt, Germany) for later viewing. In order to perform immunohistochemistry assays, the tissue sections were hydrated and placed in a glass jar containing 10 mM sodium citrate buffer, pH 6.0, and heated in a pressure cooker for 2 min. The endogenous peroxidase activity was inhibited by incubation with 3% hydrogen peroxide for 20 min at room temperature. After rinsing in Tris-buffered saline (TBS), the slides were incubated with blocking solution (3% normal donkey serum plus 0.05% Triton in TBS) for 45 min to prevent non-specific binding of the first antibody. Afterwards, the sections were incubated overnight at 4°C with the primary antibodies against proliferating cellular nuclear antigen (PCNA, Life Technologies), and Caspase 3 (Cell Signaling, Danvers, MA, USA) (1:1,000) in the blocking solution diluted 1:9. Then, the sections were washed in TBS and incubated for 20 min with biotinylated link universal antibody (Dako, Barcelona, Spain). After an extensive wash in TBS, detection was made by the conventional labeled-streptavidin-biotin method (LSAB-kit, Dako). The peroxidase activity was detected using the glucose oxidase-DAB-nickel intensification method. Sections were dehydrated, cleared in xylene, and mounted in Entellan. Sections of samples identically processed, but not incubated with the primary antibodies, were used as negative controls. As positive controls, sections of skin, rat adrenal gland and kidney were processed with the same antibody.

### Data analysis

Quantification of band densities was performed using Quantitive One Program (Bio-Rad, Alcobendas, Spain). Data were subjected to one-way ANOVA and differences were determined by Bonferroni's multiple comparison test. Each experiment was repeated at least three times. Data are the means of individual experiments and presented as mean ± SEM; *p* < 0.05 was considered statistically significant.
